# Associations between tricuspid annular plane systolic excursion to reflect right ventricular function and acute kidney injury in critically ill patients: a SICS-I sub-study

**DOI:** 10.1186/s13613-019-0513-z

**Published:** 2019-03-13

**Authors:** Renske Wiersema, Jacqueline Koeze, Bart Hiemstra, Ville Pettilä, Anders Perner, Frederik Keus, Iwan C. C. van der Horst, SICS Study Group

**Affiliations:** 1 Department of Critical Care, University Medical Center Groningen, University of Groningen, Groningen, The Netherlands; 2 Division of Intensive Care Medicine, Department of Anesthesiology, Intensive Care and Pain Medicine, University of Helsinki and Helsinki University Hospital, Helsinki, Finland; 3 Department of Intensive Care 4131, Centre for Research in Intensive Care, Copenhagen University Hospital, Rigshospitalet, Copenhagen, Denmark

**Keywords:** Prospective study, Hemodynamics, Acute kidney injury, Ultrasonography, Critical care

## Abstract

**Background:**

Acute kidney injury (AKI) occurs in up to 50% of all critically ill patients and hemodynamic abnormalities are assumed to contribute, but their nature and share is still unclear. We explored the associations between hemodynamic variables, including cardiac index and right ventricular function, and the occurrence of AKI in critically ill patients.

**Methods:**

In this prospective cohort study, we included all patients acutely admitted to an intensive care unit (ICU). Within 24 h after ICU admission clinical and hemodynamic variables were registered including ultrasonographic measurements of cardiac index and right ventricular function, assessed using tricuspid annular plane systolic excursion (TAPSE) and right ventricular systolic excursion (RV S’). Maximum AKI stage was assessed according to the KDIGO criteria during the first 72 h after admission. Multivariable logistic regression modeling was used including both known predictors and univariable significant predictors of AKI. Secondary outcomes were days alive outside ICU and 90-day mortality.

**Results:**

A total of 622 patients were included, of which 338 patients (54%) had at least AKI stage 1 within 72 h after ICU admission. In the final multivariate model higher age (OR 1.01, 95% CI 1.00–1.03, for each year), higher weight (OR 1.03 CI 1.02–1.04, for each kg), higher APACHE IV score (OR 1.02, CI 1.01–1.03, per point), lower mean arterial pressure (OR 1.02, CI 1.01–1.03, for each mmHg decrease) and lower TAPSE (OR 1.05, CI 1.02–1.09 per millimeter decrease) were all independent predictors for AKI in the final multivariate logistic regression model. Sepsis, cardiac index, RV S’ and use of vasopressors were not significantly associated with AKI in our data. AKI patients had fewer days alive outside of ICU, and their mortality rate was significantly higher than those without AKI.

**Conclusions:**

In our cohort of acutely admitted ICU patients, the incidence of AKI was 54%. Hemodynamic variables were significantly different between patients with and without AKI. A worse right ventricle function was associated with AKI in the final model, whereas cardiac index was not.

**Electronic supplementary material:**

The online version of this article (10.1186/s13613-019-0513-z) contains supplementary material, which is available to authorized users.

## Background

Acute kidney injury (AKI) occurs in up to 50% of critically ill patients and is associated with increased mortality and morbidity [1–4]. Patients with more severe AKI have increased mortality rates and even when recovered remain at increased risk of unfavorable long-term outcome [4–7]. No specific interventions to reverse AKI exist, and current guidelines make recommendations against most interventions that have been studied, suggesting to limit fluid overload and to target mean arterial blood pressure at 65 mmHg to protect renal function [8, 9].

A variety of pathophysiological mechanisms are hypothesized to be related to the development of AKI, but causal mechanisms remain largely unclear. Sepsis and hypovolemia are hypothesized to play a role, and both may result in inflammation and hypoperfusion [10, 11]. However, studies on kidney hypoperfusion suggest that reduced arterial blood flow is not the largest contributing hemodynamic factor for the development of AKI [10, 12]. In patients with sepsis renal hyperperfusion may occur and inflammatory mechanisms may dominate in AKI [11]. Another factor suggested to be associated with development of AKI is liberal fluid therapy [13–16]. Fluids may hypothetically induce venous congestion which may reduce renal blood flow and contribute to the occurrence of AKI [17]. Singular proxies of venous congestion such as fluid balance and central venous pressure (CVP) have been shown to be associated with AKI, but no definition for venous congestion exists [17, 18]. Venous congestion as contributor to AKI might also explain why fluid resuscitation does not improve kidney function [13–15, 19].

One method to advance our understanding of this complex syndrome is to explore associations between AKI development and hemodynamic variables, including cardiac index and right ventricular function in acutely admitted critically ill patients. The objective of the current sub-study was to evaluate the association of AKI with hemodynamic variables including critical care ultrasonography (CCUS) measurements of both the left and right ventricles. We hypothesized that decreased right ventricular function is associated with venous congestion. Venous congestion may cause changes in venous outflow of the kidney, in addition to the arterial component of kidney perfusion, and may be associated with the development and severity of AKI.

## Methods

### Design and setting

This is a sub-study of the Simple Intensive Care Studies I (SICS-I), a single-center, prospective observational study designed to evaluate the diagnostic and prognostic value of combinations of clinical and hemodynamic variables in critically ill patients (NCT02912624) [20, 21]. This sub-study, which we will from now on refer to as the current study, was implemented in the SICS-I on February 10, 2016. The local institutional review board (Medisch Ethische Toetsingscommissie of the University Medical Center Groningen; UMCG) approved the study (M15.168207). We report our study in adherence to STROBE guideline (Additional file 1: Table S1) [22].

### Participants and study size

All acutely admitted patients of 18 years and older with an expected ICU stay of at least 24 h were eligible for inclusion. Exclusion criteria were discharge within 24 h and absence of informed consent. In the present study, we further excluded patients in which we were unable to obtain measurements of right ventricular function and patients with history of chronic kidney disease or dialysis prior to admission. Study size was dependent on patients included within SICS-I and amount of available data.

### Variables

We registered patient characteristics at admission, including the APACHE IV scores and use of vasopressors and/or inotropes according to our protocol [23]. Variables necessary for the diagnosis (and severity) of AKI were recorded up until 72 h after admission. The hemodynamic variables were measured once (within first 24 h of ICU admission) at the earliest possibility. We prospectively recorded data from physical examination, including heart rate, systolic blood pressures (SBP), diastolic blood pressures (DBP), central venous pressure (CVP), mean arterial pressure (MAP) and central temperature. We recorded several CCUS variables (Vivid-S6, GE Healthcare, London, UK) including cardiac index, tricuspid annular plane systolic excursion (TAPSE) and right ventricular systolic excursion (RV S’). Cardiac index was estimated by the velocity time integral of left ventricular outflow tract times heart rate times left ventricular outflow tract area (cardiac output = VTI × HR × LVOT area), divided by body surface area. All measurements were conducted by research-students, who were not involved in patient care. All researchers were trained by experienced cardiologist-intensivists to conduct a focused CCUS before contributing to the study. Training included studying of theory, practice on healthy individuals and lastly supervised CCUS in patients. The first 20 examinations were supervised by a senior researcher. The images and measurements of cardiac index were validated and remeasured by an independent core laboratory (Groningen Image Core Lab, UMCG, Groningen, the Netherlands) for purposes of the main study.

### Definitions

We defined AKI and its severity according to the KDIGO criteria based on serum creatinine, urinary output recorded cumulative every 24 h and use of RRT [24]. The modification of diet in renal disease (MDRD) formula was used for estimation of the ideal serum creatinine for each individual as baseline assuming a creatinine clearance of 75 ml/min/m^2^, as suggested by [25, 26]. Chronic kidney disease was defined by serum creatinine above 177 µmol/L (following the definition used by the ‘Nationale Intensive Care Evaluatie’) [27]. Cutoff values for cardiac function were below 2.2 L/min/m^2^ for low cardiac index, below 17 mm for low TAPSE and below 9.5 cm/s as low RV S’ [28]. Patient outcome variables were the occurrence and maximum severity of AKI within the first 72 h of ICU stay, number of days alive outside ICU and 90-day mortality.

### Bias

To assess selection bias we compared baseline characteristics between included and excluded patients. We aimed to minimize possible misclassification bias by providing comprehensive protocols and training for involved researchers.

### Statistics

The overall statistical methods were described in the pre-defined statistical analysis plan of SICS-I (NCT02912624). Continuous variables were reported as means [with standard deviations (SD)] or medians [with interquartile ranges (IQR)] depending on distribution. Categorical data were presented in proportions. Associations were calculated as odds ratios (OR) with 95% confidence intervals (CI). Student’s *T* test, Mann–Whitney *U* test or the Chi-square tests were used as appropriate. A two-sided *p* value of ˂0.05 was considered statistically significant. Associations of hemodynamic variables with the occurrence of AKI were first explored by univariate analysis. We used univariate associations with *p* < 0.1 for entrance of a variable into the multivariable model, while the predisposing variables age and sepsis were included in the model based on the literature, irrespective of their *p* values in the univariate analyses. No missing data were imputed, and a multivariate analysis was performed based on available original data, except for variables with more than 50% missing values, which were excluded from the model [23]. Variables were assessed for collinearity before entrance in the multivariable model. If two variables were strongly correlated, only one variable was included in the multivariate model based on the strongest univariate association. The final model was based on logistic regression analysis to identify the variables independently associated with the development of AKI. Discrimination (to distinguish AKI patients from non-AKI patients) of the final model was evaluated with receiver operating characteristic (ROC) curves. Calibration of the multivariable model (i.e., the number of AKI cases that the model predicts correctly across different risk groups) was checked with the Hosmer–Lemeshow test and by plotting observed AKI proportions against predicted risks of 10 equally sized groups. Analyses were performed using Stata, version 15 (StataCorp, College Station, TX, USA).

## Results

**Fig. 1:**
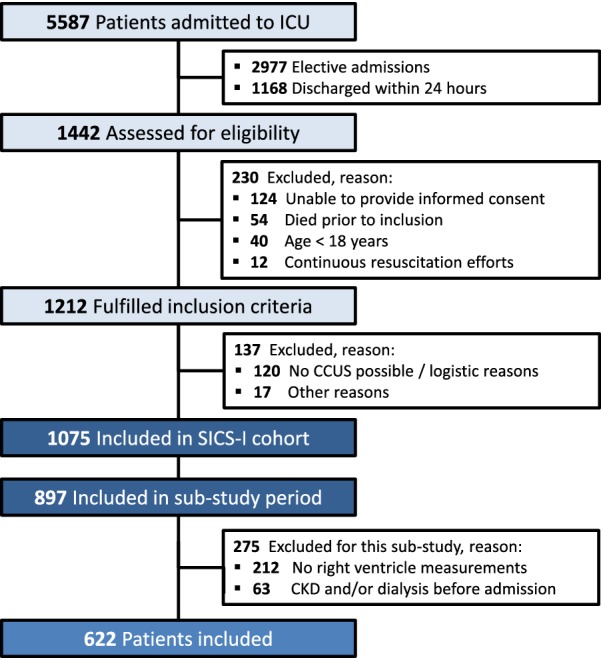
Study inclusion flowchart

Between March 27, 2015, and July 22, 2017, a total of 1075 patients were included in the SICS-I study. The present study started on February 10, 2016, and included 897 patients. A total of 63 patients had a history of CKD or dialysis prior to ICU admission and were therefore excluded. In 212 patients, CCUS did not include right ventricular assessment, leaving 622 patients to be included (Fig. 1). Median time to inclusion was 14 (± 8) h after ICU admission. We evaluated the baseline characteristics of the included patients and the 212 patients excluded due to missing values of right ventricle assessment. There was significantly higher weight, lower percentage of diabetic patients, more use of vasopressors and percentage of mechanical ventilation in the excluded group; other baseline variables and APACHE IV score were similar (Additional file 1: Table S2).

A total of 338 patients (54%) fulfilled the criteria for AKI within 72 h after admission, including 105 patients (17%) with AKI stage 1; 136 patients (22%) with AKI stage 2; and 97 patients (16%) with AKI stage 3, respectively. One patient with AKI stage 1 could not be evaluated for deterioration into AKI stage 2 or 3 due to early death. Several baseline characteristics of patients with and without AKI were different (Table 1). The mean observed cardiac index was 2.6 (± 0.9) L/min/m^2^; mean TAPSE was 19.5 (± 6.1) mm; and mean RV S’ was 13.5 (± 4.1) cm/s. Low cardiac index, low TAPSE and low RV S’ were observed in 35%, 33% and 16% of patients, respectively. Of the 338 patients with AKI, 61 patients had both low CI and low TAPSE, compared to 19 patients in the non-AKI group (*p* = 0.001). RRT was instigated in 32 patients (5.1%) during their ICU stay. No loss to follow-up occurred. Median days alive out of ICU at 90-day follow-up were 86.6 days (IQR 78.2–88.2) in patients without AKI and 83.5 days (IQR 3.0–87.4) in patients with AKI (*p* < 0.001). Of the 622 patients, 161 patients (26%) had died at 90-day follow-up. Mortality was 33% in the AKI group and 18% in the non-AKI group (*p* < 0.001).

**Table 1:** Baseline and hemodynamic characteristics in the overall population and patients with and without AKI

	Total (*n *= 622)	No AKI (*n *= 277)	AKI (*n *= 376)	*p* value *
Age, years (SD)	61 (15)	58 (16)	64 (14)	< 0.001
Gender, *n* male (%)	385 (62%)	180 (63%)	205 (61%)	0.49
Height, cm (SD)	175.6 (9.5)	175.8 (9.9)	175.5 (9.1)	0.71
Weight, kg (SD)	81.6 (16.5)	77.9 (14.9)	84.7 (17.1)	< 0.001
Diabetes mellitus, *n* (%)	129 (21%)	57 (20%)	72 (21%)	0.71
Liver cirrhosis, *n* (%)	21 (3.4%)	7 (2.5%)	14 (4.1%)	0.25
Mechanical ventilation, *n* (%)	357 (57%)	159 (56%)	198 (59%)	0.51
PEEP, cm H_2_O (IQR)	7.0 (5.0, 8.0)	6.0 (5.0, 8.0)	8.0 (5.0, 10.0)	< 0.001
Sepsis, *n* (%)	100 (16%)	38 (13%)	62 (18%)	0.093
Use of vasopressors, *n* (%)	287 (46%)	108 (38%)	179 (53%)	< 0.001
Use of RRT, *n* (%)	32 (5.1%)	0 (0.0%)	32 (9.5%)	< 0.001
APACHE IV, mean (SD)	74.9 (28.9)	64.6 (23.4)	83.6 (30.2)	< 0.001
*Hemodynamic variables*				
Heart rate, bpm (SD)	84 (20)	81 (19)	87 (20)	< 0.001
Mean arterial pressure, mmHg (SD)	79 (15)	83 (15)	77 (15)	< 0.001
Systolic BP, mmHg (SD)	120 (26)	126 (26)	115 (24)	< 0.001
Diastolic BP, mmHg (SD)	60 (12)	62 (11)	59 (12)	0.007
Cardiac index, L/min/m^2^ (SD)	2.7 (0.9)	2.8 (0.9)	2.6 (1.0)	0.026
Central temperature, °C (SD)	36.9 (0.9)	37.0 (0.9)	36.9 (0.9)	0.019
CVP, mmHg (SD)	9.1 (5.7)	7.3 (4.6)	10.1 (6.1)	0.015
RV S’, cm/s (SD)	13.5 (4.1)	14.1 (3.7)	13.1 (4.3)	0.002
TAPSE, mm (SD)	19.5 (6.1)	20.9 (5.5)	18.4 (6.4)	< 0.001

*SD* standard deviation, *PEEP* positive end-expiratory pressure, *RRT* renal replacement therapy, *CVP* central venous pressure, *RV S*’ right ventricular systolic excursion, *TAPSE* tricuspid annular plane systolic excursion

*p value of difference between non-AKI and AKI patients

### Associations with AKI

All hemodynamic variables were statistically significantly associated with AKI in the univariate analysis (Additional file 1: Table S3). CVP was missing in over 50% of cases (measured in only 112 patients) and thus was not included in the final multivariate model. Due to collinearity between MAP and systolic and diastolic blood pressures, we only included MAP in the model. No further collinearity was observed. All remaining variables with *p* < 0.1 in univariate analysis were entered into the adjusted model.

In multivariate analysis, positive end-expiratory pressure (PEEP), cardiac index, RV S’, vasopressors and central temperature were shown to have a *p* > 0.1 and were removed from the model (*p* = 0.95, *p* = 0.71, *p* = 0.19, *p* = 0.17 and *p* = 0.10, respectively). In the final model heart rate was not significantly associated with AKI. Higher age, higher weight, increased APACHE IV scores, lower MAP and lower TAPSE were all statistically significantly associated with AKI (Table 2). The receiver operating characteristic (ROC) curve of the final model yielded an area under the curve of 0.75 (95% CI 0.73–0.80), pseudo-*R*^2^ = 0.15. Hosmer–Lemeshow goodness-of-fit test showed a *χ*^2^ 10.04; *p* = 0.2623.

**Table 2:** Variables independently associated with the development of AKI in multivariable analysis

	OR	95% CI		*p* value
Age (per year increase)	1.01	1.00	1.03	0.038
Weight (per kg increase)	1.03	1.02	1.04	0.000
APACHE IV (per point increase)	1.02	1.01	1.03	0.000
Mean arterial pressure (per mmHg decrease)	1.02	1.01	1.03	0.001
TAPSE (per mm decrease)	1.05	1.02	1.08	0.002

Description: pseudo-*R*^2^ = 0.15. Hosmer–Lemeshow goodness-of-fit test *χ*^2^ 10.04; *p* = 0.2623. AUC = 0.75 (95% CI 0.73–0.80). *APACHE* acute physiology and chronic health evaluation; *TAPSE* tricuspid annular plane systolic excursion

A sensitivity analysis was performed with the final model and CVP in patients in which this was available (*n* = 94), which showed no statistically significant association for CVP (OR 1.08; 95% CI 0.99–1.20, *p* = 0.098) or lower TAPSE (OR 1.04; 95% CI 0.96–1.13, *p* = 0.252) with AKI (Table 3).

**Table 3:** Sensitivity analysis with central venous pressure in the multivariate model (*n* = 94)

	OR	95% CI		*p* value
Age (per year increase)	1.00	0.96	1.04	0.898
Weight (per kg increase)	1.04	1.01	1.07	0.006
APACHE IV (per point increase)	1.02	1.00	1.04	0.049
Mean arterial pressure (per mmHg decrease)	1.01	0.96	1.05	0.659
TAPSE (per mm decrease)	1.05	0.96	1.12	0.252
CVP (per mmHg increase)	1.08	0.98	1.18	0.098

## Discussion

In this prospective observational study, we found that lower MAP and lower TAPSE were both independently statistically significantly associated with the development of AKI, along with higher age, higher weight and increased APACHE IV scores. Cardiac index was not independently statistically significantly associated with AKI.

### Venous congestion and AKI

Singular proxies of venous hemodynamic function have previously been associated with the development of AKI [18, 29–33]. Chen et al. found that peripheral edema was associated with a 30% higher risk of AKI in 2238 critically ill patients. In the same article the authors showed that each cm H_2_O higher CVP was associated with a 2% higher adjusted risk of AKI in 4761 critically ill patients [17]. We assessed right ventricular function measured by TAPSE and confirmed these observations of the potential role of central venous hemodynamic function, assuming that venous congestion, with CVP as a proxy, leads to impaired right ventricular function, and vice versa. In the sensitivity analysis including CVP the point estimates were similar, but in a relatively small sample size these effects did not reach statistical significance. Right ventricular function has also been suggested to be associated with the development of AKI by data from patients with heart failure by Mullens et al. [34] and pulmonary hypertension by Haddad et al. [35]. Guven et al. [36] described that preoperative right ventricular function was associated with an increased risk and severity of AKI in 595 heart transplant patients. To our best knowledge, in no studies these measurements were performed prospectively in an unselected population of the critically ill. We evaluated two measures of right ventricular function: TAPSE and RV S’. Huang et al. [37] elegantly described that most studies use these as indices of RV function, but evaluation of isolated TAPSE is potentially misleading. Other indices of RV function or for example inferior vena cava measurements may also reflect venous congestion and be associated with AKI [38]. Fluid balance may influence right ventricular function and thus interact with the association with AKI [39]. The optimal variable representing venous hemodynamic function for evaluation of association with AKI in the critically ill is to be elucidated. A combination of previously mentioned variables might show a higher predictive value for venous congestion and AKI in critically ill patients compared to TAPSE alone.

### Arterial function and AKI

Only limited data specifically address the association between cardiac index and AKI. Many studies focus on the association between (septic) shock and AKI [40, 41], while data on the detailed association between cardiac index, MAP and renal blood flow in critically ill patients in general and AKI are sparse [42, 43]. In a retrospective cohort study of 1879 critically ill patients, both impaired left and right ventricular function were associated with AKI and increased mortality [44]. One study included 10 patients with sepsis and AKI and suggested that the renal perfusion fraction of cardiac output measured by phase contrast magnetic resonance imaging was very low (7.1%) [45]. One hypothesis is that AKI develops after ischemic and hypoxic damage due to restricted blood flow, possibly caused or exaggerated by a reduced cardiac index. A higher cardiac index may be protective for AKI, or reverse, that a low cardiac index would be associated with increased incidences of AKI, although other mechanisms may be involved. We found no association between cardiac index and AKI, while a lower MAP appeared to be associated with AKI. Findings from a previous prospective observational study evaluating associations between hemodynamic variables and the progression of AKI in septic patients suggested that a lower MAP is associated with AKI [40].

Similar to other studies, higher PEEP showed association with AKI; however, in the multivariate model PEEP did not remain significant [46, 47]. The fact that PEEP in this study did not contribute enough to the final model might be explained by the relatively low PEEP levels and the regularly used lower tidal volumes (for the purposes of lung-protective ventilation) leading to substantial lower intrathoracic pressures compared with the mentioned studies. Moreover, only 57% of our population was mechanically ventilated, potentially leading to a bias in our multivariate analysis in terms of this variable, and we did not evaluate other ventilation settings such as plateau pressure.

### Implications and generalizability

We conducted a single-center study. Collaboration with other centers and other ICUs will increase generalizability. We included a broad population of acutely admitted critically ill patients, while investigation of hemodynamic variables in subgroups might yield more specific clues of the role of hemodynamic variables in the pathophysiology of AKI according to diagnosis, e.g., sepsis or shock patients. Our observations encourage to elaborate specifically on the role of venous hemodynamic function in the development of AKI and to search for a practical proxy, or perhaps a combination of multiple variables, for assessment of venous congestion. This is also in line with the recently proposed research agenda for AKI [48].

### Limitations

Several limitations of our study must be acknowledged. First, our study is a prospective observational study, which hampers causal inferences. As it is a data-driven analysis, results need to be confirmed in other studies. Second, we collected kidney function up to 72 h, i.e., early AKI. Therefore, the incidence and severity of AKI may both have deteriorated or improved after 72 h. It has been suggested that the incidence of AKI is highest during the initial days of ICU stay [49], although continued fluid administration during or after early AKI might prevent recovery of kidney function [50]. Variables identified as associated with AKI might vary with the timing of assessment of variables and diagnosing AKI [51], so that longer follow-up will probably lead to identification of another set of variables associated with AKI. Third, CVP was measured in fewer patients and had to be excluded from multivariate analysis in this cohort. This is an important limitation, as CVP may serve as one of the proxies for venous congestion and right ventricular function. This, however, does not influence the conclusion that TAPSE was associated with AKI, whereas cardiac index was not. Fourth, we used the MDRD formula to estimate baseline serum creatinine since these data were not collected in the main study. Even though this method is widely accepted, this might have led to either an over- or underestimation of AKI incidence [52, 53]. Fifth, we used serum creatinine as a marker of kidney function. Despite recent observations on the accuracy of serum creatinine [26], serum creatinine is still a surrogate for kidney function. Also, creatinine might be biased by interventions during ICU stay, such as hemodilution due to a positive fluid balance. Last, 21% of the patients were excluded from analyses due to inadequate quality of CCUS images which may be difficult to obtain in ICU patients [54]. Weight and mechanical ventilation were significantly different in this group, which are factors that often hamper transthoracic CCUS. The use of vasopressors was also more prevalent in the excluded group, but as APACHE IV score was not significantly different, we assumed that the groups were similar. We thus do not expect that this has influenced the findings of this study.

## Conclusions

In acutely admitted ICU patients decreased right ventricular systolic function and MAP, but not cardiac index, were associated with the development of AKI. Further research elaborating on right heart function in combination with other proxies for venous congestion may elucidate its role in the development or worsening of AKI in the critically ill.

## Additional file

**Additional file 1.** **Table S1.** Strobe statement checklist with according the place in the manuscript. **Table S2.** Baseline characteristics in the overall population and included population. **Table S3.** Variables independently associated with the development of AKI in univariate analysis.

## Abbreviations

AKI: acute kidney injury
APACHE IV: acute physiology and chronic health evaluation
CCUS: critical care ultrasonography
CI: confidence interval
CVP: central venous pressure
ESM: electronic supplemental material
KDIGO: Kidney Disease Improving Global Outcome
MAP: mean arterial pressure
MDRD: modification of diet in renal disease
OR: odds ratio
ICU: intensive care unit
TAPSE: tricuspid annular plane systolic excursion
RRT: renal replacement therapy
RV S’: right ventricular systolic excursion
SICS-I: Simple Intensive Care Studies I
STROBE: Strengthening the Reporting of Observational Studies in Epidemiology
UMCG: University Medical Center Groningen
